# Global Public Attitudes About Clinical Research and Patient Experiences With Clinical Trials

**DOI:** 10.1001/jamanetworkopen.2018.2969

**Published:** 2018-10-05

**Authors:** Annick Anderson, Deborah Borfitz, Kenneth Getz

**Affiliations:** 1 The Center for Information and Study on Clinical Research Participation, Boston, Massachusetts; 2 Tufts Center for the Study of Drug Development, Tufts University School of Medicine, Boston, Massachusetts

## Abstract

**Importance:**

Effective, continuous improvement in patient engagement depends on an intimate understanding of public and patient perceptions and experiences in clinical research.

**Objectives:**

To identify the views of clinical trial participants and nonparticipants and characterize trends in these views over time.

**Design, Setting, and Participants:**

In this survey study, a questionnaire was administered online from May 8 to July 24, 2017, by the Center for Information and Study on Clinical Research Participation (CISCRP), and findings were compared with previous studies conducted in 2013 and 2015. The 2017 sample included responses from 12 427 individuals from 68 countries and represents a 10% participation rate. Similar to international assessments conducted by the CISCRP and other organizations, this study drew responses from a convenience sample; any adult older than 18 years who received an email or had online access was eligible to participate.

**Main Outcomes and Measures:**

Significant changes were observed in the perceptions and clinical trial experiences of the public and study volunteers compared with past CISCRP studies.

**Results:**

A total of 12 427 individuals (mean [SD] age, 55 [15] years; 7355 [59.2%] female; 10 085 [81.2%] white), 2194 (17.7%) of whom had participated in previous clinical research studies, responded to the survey in 2017. Findings indicated a belief in the importance of clinical research, but limited understanding of the research process persists. In 2017, a total of 10 506 individuals (84.5%) perceived clinical research to be very important to the discovery and development of new medicines; however, 4079 of 6919 respondents (59.0%) were unable to name a place where studies were conducted. A total of 11 182 respondents (90.0%) believed that clinical research is generally safe; however, 5578 of 12 427 individuals (44.9%) reported that clinical trials are rarely considered as an option when discussing treatments or medications with their physician. Clinical trial participation was perceived as inconvenient and burdensome; in the latest survey, 1075 respondents (49.0%) expressed that their clinical trial participation disrupted their daily routine.

**Conclusions and Relevance:**

The results of this study may provide a foundation from which to build meaningful and effective engagement with the public and patients and revealed roadblocks, including knowledge gaps among the public, limited physician involvement in discussing clinical trials as treatment options, and the inconveniences that patients encounter after they volunteer to participate. These findings may inform patient engagement strategies and tactics and ultimately help accelerate the drug-development process.

## Key Points

**Question:**

How have the perceptions of clinical research among the public and clinical trial participants evolved over time?

**Findings:**

This survey study of 12 427 individuals (representing 68 countries and including 2194 clinical trial participants) responding to a questionnaire in 2017 revealed a perceived importance of clinical research but continued limited understanding of the clinical research process, that clinical trials are rarely discussed during regular physician visits, and that clinical trial participation is perceived as burdensome.

**Meaning:**

These findings may inform patient engagement strategies that may ultimately accelerate the pace at which useful medicines are developed and commercialized.

## Teaser

This survey study reviews the findings of the attitudes, perceptions, and experiences of clinical trials among clinical trial participants and nonparticipants in 68 countries.

## Introduction

Stakeholders in the clinical research enterprise have become keenly interested in improving public and patient engagement in clinical trials^1^ for reasons that include ensuring that the most relevant and clinically meaningful outcomes are being assessed and improving study volunteer recruitment and retention rates.^2^ Increasing protocol design complexity during the past 2 decades has had an adverse effect on the cost and length of the drug-development process, placed undue burden on clinical research professionals administering clinical trial procedures,^3,4^ and impeded the willingness of study volunteers to be screened for and remain in a study through its completion.^4^

Among initiatives being piloted and implemented are the following: (1) US Food and Drug Administration and European Medicines Agency meetings with patients and their families to learn about direct experiences managing select diseases; (2) pharmaceutical and biotechnology company collaborations with patient advocacy groups and social media patient communities; (3) the use of patient advisory board panels to solicit input on draft protocol designs; (4) the deployment of telemedicine and home nursing networks to improve participation convenience; and (5) the return of clinical trial results summaries in plain language to study volunteers.^5,6^ Government and industry research sponsors have increasingly recognized the important role of health care professionals as patient engagement facilitators. Patients look to their physicians and nurses as the most trusted and primary source for education and information about clinical trials and for advice on whether to enroll in a clinical trial.^7,8^ As the volume of clinical research activity within clinical care settings increases, health care professionals are expected to play a larger role in identifying and assisting patient participants.^9^

Effective, continuous improvement in patient engagement depends on an intimate understanding of public and patient attitudes and perceptions and experiences in clinical research. It has been more than 12 years since public knowledge and perceptions of clinical research were formally discussed in *JAMA*,^10^ and at that time, only the opinions of Americans were summarized.

In this article, we present key findings of a 2017, multicontinent (68-country) survey examining attitudes, perceptions, and experiences of clinical research to provide updated benchmarks that inform patient engagement strategies and tactics implemented by clinical research and clinical care professionals. This study examined the views of clinical trial participants and nonparticipants over time to provide insight into gaps in public awareness and knowledge about clinical research, identify opportunities to reduce unnecessary participation burden, and reveal where current efforts are or are not resonating with the public and patients. Continuous assessment of public and patient attitudes, perceptions, and experiences in clinical research may inform patient engagement strategies and tactics that may ultimately accelerate the pace at which useful medicines are currently getting to market and help mitigate increasing drug-development costs and decreasing success rates of new drug and biologic agent approvals.^11,12^

## Methods

The Center for Information and Study on Clinical Research Participation (CISCRP), an independent nonprofit organization in Boston, Massachusetts, began administering a global, online study on a biennial basis in 2013 to evaluate public and patient perceptions about clinical research and the motivations and experiences of clinical trial participants. This study followed the American Association for Public Opinion Research (AAPOR) reporting guideline. Implied consent was used in the 2017 study. Participants were informed of the study objectives and risks and benefits of the survey and assured of anonymity. As an incentive, participants were offered entry into a gift card drawing of $10.00 (or local currency equivalent) on completion of the survey. The 2017 study was reviewed and deemed to be exempt by the New England Institutional Review Board, a division of the Western Institutional Review Board. All data were deidentified.

### Study Participants and Data Collection

Survey responses were collected online from May 8 to July 24, 2017, with the support of organizations that included Acurian, Clariness, CureClick, HealthUnlocked, and IQVIA. These organizations disseminated an online link to the survey to their respective networks. These networks were composed of individuals who have opted to receive health-related information. Race/ethnicity of the study participants was defined by the respondents, although they were not required to provide this information. The collection of race/ethnicity data was important to identify various trends among specific populations.

The findings are organized into 3 distinct groupings: those in the community at large,^13^ those actively searching for clinical trials or expressing interest in clinical trials,^14^ and those participating in and completing a clinical trial,^15^ The findings from these groups were compared with those of similar cohorts of patients responding to the CISCRP 2013^16,17,18^ and 2015^19,20,21^ survey results.

### Survey Instrument

The survey instrument was developed by a cross-functional work group composed of patients and representatives from biopharmaceutical companies, contract research organizations, and investigative sites. The instrument was translated into Spanish, German, Russian, Chinese, and Japanese.

To allow for longitudinal analyses, many of the questions on the original 2013 survey were posed in subsequent surveys in 2015 and 2017. Additional, new topic areas were incorporated in the latest survey to provide baseline figures for future comparisons about issues of heightened recent interest, including the association between health care professionals and the clinical research experience and initiatives that enhance study volunteer convenience, enrollment, and retention. The overall structure of the survey has remained constant, starting with questions that assess general clinical research impressions among the public and patients using conditional branching to further probe those who had actively searched for trials, expressed an interest in doing so, or participated in a clinical trial.

### Statistical Analysis

The 2017 survey was programmed in and administered via an online survey tool. The 2017 data set was subsequently exported from the online survey tool and imported into MarketSight data analysis and visualization software (MarketSight LLC). Data sets for the 2013 and 2015 studies were also imported into this software. Comparisons among subgroups in the 2017 study and with the 2013 and 2015 studies were performed using *z* tests, and comparisons of means were performed using *t* tests. Tests were conducted at the 95% confidence level. All tests were 2-sided, with *P* < .05 considered to be statistically significant. The data for this article were specifically chosen to reveal multiyear trends in public and patient perceptions about the value of clinical research, factors that influenced their decision whether to participate in a study, and the clinical trial experience.

## Results

A total of 12 427 individuals (mean [SD] age, 55 [15] years; 7355 [59.2%] female; 10 085 [81.2%] white), 2194 (17.7%) of whom had participated in previous clinical research studies, responded to the survey in 2017. Of the 12 427 respondents, 7991 (64.3%) had been diagnosed with a medical condition. The survey was distributed to approximately 120 400 individuals globally, representing a participation rate of 10%. Table 1 summarizes the 2017 survey respondent characteristics and compares them with those from the 2015 and 2013 surveys. The concentrations of respondents in 2017 were 5693 (45.8%) from North America, 3541 (28.5%) from Europe, 1699 (13.7%) from the Asia-Pacific region, 915 (7.4%) from South America, and 579 (4.7%) from Africa.

**Table 1.** Respondent Profiles

Characteristic	No. (%) of Respondents		
	2013 (n = 5701)^a^	2015 (n = 12 009)^b^	2017 (n = 12 427)^c^
Sex			
Male	2393 (42.4)	5509 (45.9)	4945 (39.8)
Female	3254 (57.6)	6500 (54.1)	7355 (59.2)
Region			
North America	4286 (75.3)	6665 (55.5)	5693 (45.8)
South America	233 (4.1)	877 (7.3)	915 (7.4)
Europe	837 (14.7)	2618 (21.8)	3541 (28.5)
Asia Pacific	329 (5.8)	1302 (10.8)	1699 (13.7)
Africa	10 (0.1)	547 (4.6)	579 (4.7)
Age, y			
18-34	1027 (18.0)	2279 (19.0)	1624 (13.1)
35-44	969 (17.0)	1766 (14.7)	1409 (11.3)
45-54	1311 (23.0)	2495 (20.8)	2396 (19.3)
55-64	1482 (26.0)	2929 (24.4)	3388 (27.3)
≥65	912 (16.0)	2540 (21.2)	3601 (29.0)
Race (top mentions)			
White	NA	9905 (82.5)	10 085 (81.2)
Black	NA	802 (6.7)	695 (5.6)
Asian	NA	551 (4.6)	691 (5.6)
Ethnicity (top mentions)			
Non-Hispanic/Latino	NA	10016 (83.4)	10 936 (88.0)
Hispanic/Latino	NA	949 (7.9)	958 (7.7)
Educational level			
No school or primary	0	138 (1.1)	234 (1.9)
Some or completed high school	1112 (20.0)	2851 (23.7)	3002 (24.2)
Some or completed college	3515 (61.7)	7448 (62.0)	7205 (58.0)
Completed postgraduate work	1074 (18.8)	1572 (13.1)	1986 (16.0)

Abbreviation: NA, not asked.

^a^ Data are from the 2013 Center for Information & Study on Clinical Research Participation Perceptions and Insights Study. Some columns may not total 100% because other responses are not shown. Percentages not all based on 5701 because of missing responses to some survey items (n = 5647 for sex and 695 for region).

^b^ Data are from the 2015 Center for Information & Study on Clinical Research Participation Perceptions & Insights Study. Some columns may not total 100% because other responses are not shown.

^c^ Data are from the 2017 Center for Information & Study on Clinical Research Participation Perceptions & Insights Study. Some columns may not total 100% because other responses are not shown.

### Perceptions and Understanding

Among the general public and patients at large, 10 506 (84.5%) in 2017 perceived clinical research to be very important to the discovery and development of new medicines. This result is similar to the findings of the 2 earlier CISCRP surveys. Although interest in participating in a clinical trial remains high overall, a smaller proportion of individuals (3848 [31.0%]) were very willing in 2017 compared with prior studies, and 4794 (38.6%) were not confident that they could find an appropriate clinical trial. Both of these figures were significantly higher for those who had participated in a clinical research study in the past and were more adept at using online clinical trial registries to locate a trial (general willingness to participate: 1296 of 2194 clinical trial participants [59.1%] vs 2552 of 10 233 nonparticipants [24.9%]; confidence finding trial: 432 of 2194 clinical trial participants [19.7%] vs 4362 of 10 233 nonparticipants [42.6%]).

The public’s understanding of clinical research overall remained highly limited. In the 2017 study, 4079 of 6919 respondents (59.0%) were unable to name a place where studies were conducted. An even higher number (4579 of 6919 [66.2%) in the 2017 survey could not name an agency that oversees the safety of clinical research. The time to bring a drug to market (a mean of 12 years) also continued to be underestimated. In the 2017 survey, nearly 4868 respondents (39.2%) indicated that they believed the entire drug-development process takes less than 5 years. A total of 4068 respondents (32.7%) correctly noted that the drug-development cycle takes 6 to 10 years.

### Safety Concerns

Most of the public believed that clinical research is generally safe (11 182 [90.0%]). Among the 1244 respondents (10.0%) who had safety concerns, the possibility of adverse effects was the top reason mentioned (805 [64.7%]), followed by distrust of pharmaceutical companies (458 [36.8%]).

The top perceived risks and benefits associated with clinical research remained largely unchanged over time (Table 2), with adverse effects (4979 [40.1%]) and worsened overall health (4097 [33.0%]) still leading the list of concerns in 2017. Altruistic variables, such as helping to advance science (3266 [26.3%]) and helping others (3213 [25.9%]), remained the most named benefits of participation, as they were in earlier surveys.

**Table 2.** Top Perceived Benefits and Risks to Clinical Research Participation

Benefits and Risks	No. (%) of Respondents		
	2013 (n = 5701)^a^	2015 (n = 12 009)^b^	2017 (n = 12 427)^c^
**Top Mentioned Benefits **			
May help advance science and the treatment of my disease or condition	1848 (32.6)	3400 (28.3)	3266 (26.3)
May help save or improve the lives of other patients	1638 (28.9)	3126 (26.0)	3213 (25.9)
May help improve my disease or condition	831 (14.7)	2052 (17.1)	1895 (15.2)
May represent the best treatment option	NA	NA	1016 (8.2)
May provide monetary compensation for participation	286 (5.1)	598 (5.0)	592 (4.8)
May guide understanding of how available medications compare with a new treatment	384 (6.8)	780 (6.5)	545 (4.4)
May receive more care and attention from physicians and staff	148 (2.6)	650 (5.4)	507 (4.1)
**Top Mentioned Risks**			
Possibility of adverse effects	3206 (56.7)	5180 (43.1)	4979 (40.1)
Possible risks to my overall health	1145 (20.3)	3086 (25.7)	4097 (33.0)
Possibility of receiving a placebo or inactive drug	722 (12.8)	1311 (10.9)	876 (7.0)
Possibility of stopping treatments that may be providing some benefit	NA	1247 (10.4)	917 (7.4)
Possibility of making my private medical information public	260 (4.6)	471 (3.9)	314 (2.5)
Possibility of missing too much time at work	NA	NA	233 (1.9)

Abbreviation: NA, not asked (in 2013 and/or 2015 studies).

^a^ Data are from the 2013 Center for Information & Study on Clinical Research Participation Perceptions & Insights Study (all respondents). Percentages not all based on 5701 because of missing responses to some survey items (n = 5669 in the benefits sample and 5650 in the risks sample).

^b^ Data are from the 2015 Center for Information & Study on Clinical Research Participation Perceptions & Insights Study (all respondents).

^c^ Data are from the 2017 Center for Information & Study on Clinical Research Participation Perceptions & Insights Study (all respondents).

### Physician Involvement vs Patient Interest

Consistent with past CISCRP studies, most survey respondents in 2017 (9258 [74.5%]) indicated a willingness to participate in a clinical trial. However, 5578 (44.9%) also reported that clinical trials are rarely considered as an option when discussing treatments or medications with their physician. In the 2017 study, 11 719 respondents (94.3%) thought that it was important that their physician be aware of studies being conducted in their community. A well-informed health care professional was also found to positively affect patient perceptions of clinical research and participation experiences. A total of 11 545 patients (92.9%) reported being somewhat to very comfortable with having their health records used to identify an appropriate clinical trial collaboratively with their physician. A high comfort level among patients in using the health information to identify clinical trials is in contrast with a recent study^22^ that found patient distrust in medical leaders and the health system as a whole.

In each of the 3 CISCRP studies, patients consistently reported a preference to learn about clinical research through their regular physician or specialist more than any other source. This preference has increased (2963 [52.0%] in 2013, 6146 [51.0%] in 2015, and 7893 [63.5%] in 2017) and is especially true for older patients (Table 3). In the 2017 study, the highest number of the 2194 study volunteers (426 [19.4%]) reported learning about clinical trial opportunities from their physician, followed by a research center investigator or study staff (386 [17.6%]) and patient recruitment advertisements (349 [15.9%]) (eFigure 1 in the Supplement).

**Table 3.** Best Ways to Learn About Clinical Research as Reported by Age (2017)^a^

Learning Method	No. (%) of Respondents by Age Subgroup					
	18-34 y (n = 1624)	35-44 y (n = 1409)	45-54 y (n = 2396)	55-64 y (n = 3388)	≥65 y (n = 3610)	*P* Value^b^
Discussions with physician	823 (50.7)	777 (55.1)	1450 (60.5)^c^	2295 (67.7)^c^	2548 (70.6)^c^	<.001
Information at physician’s office	799 (49.2)	701 (49.8)	1296 (54.1)	1985 (58.6)^c^	2135 (59.1)^c^	<.001
Educational program at hospital	693 (42.7)^c^	564 (40.0)^c^	859 (35.9)	1236 (36.5)^c^	1158 (32.1)	<.001
Educational program at school	526 (32.4)^c^	282 (20.0)^c^	380 (15.9)^c^	461 (13.6)	409 (11.3)	<.001

^a^ 2017 Center for Information & Study on Clinical Research Participation Perceptions & Insights Study (all respondents).

^b^ The *z* test *P* value for age variable was corrected for type I error, and the test results were adjusted by multiplying the *P* value for each test by the *df*.

^c^ Statistical significance at 95% compared with the other age group(s).

Among patients in general, 10 970 (88.3%) reported that they would value being informed of clinical research opportunities during their regular physician visits. Only 502 of 2194 respondents (22.9%) reported that this had happened. A total of 11 256 respondents (90.6%) also indicated that it would be very convenient if clinical research procedures could be performed during regular physician visits. Integrating routine and clinical study visits appealed disproportionately more to women than to men.

### Multifaceted Decision Making

The choice to participate in a clinical trial is not straightforward but involves numerous considerations. In the 2017 survey, choice to participate depended on the potential risks and benefits of the study (10 264 [82.6%]), the purpose of the study (9269 [74.6%]), and the types of medical procedures required by the protocol (9063 [72.9%]) (Table 4). More practical aspects were also identified as important, including the physical location of the study center (7429 [59.8%]), potential costs and reimbursements (7106 [57.2%]), the length of participation (7019 [56.5%]), and receipt of a study summary (6979 [56.2%]).

**Table 4.** Most Important Participation Factors

Participation Factor	No. (%) of Respondents Rating Very Important	
	2015 (n = 12 009)^a^	2017 (n = 12 427)^b^
Potential risks and benefits	9059 (75.4)	10 264 (82.6)
Purpose of the clinical research study	8263 (68.8)	9269 (74.6)
Types of medical procedures required^c^	6374 (53.1)	9063 (72.9)
If my confidentiality would be protected	6745 (56.2)	7783 (62.6)
Physical location of the research study center	6591 (54.9)	7429 (59.8)
Potential costs and reimbursements	5918 (49.3)	7106 (57.2)
Length of participation	5836 (48.6)	7019 (56.5)
Receiving a summary of the study results after my participation ended	6270 (52.2)	6979 (56.2)
Being provided with supporting information on the clinical research study	NA	6703 (53.9)
Provided with information on managing my health condition in general	NA	6580 (52.9)
Duration of each study visit	NA	6157 (49.5)
No. of study visits^c^	6374 (53.1)	5931 (47.7)
If I would have access to the study drug after my participation ended	5006 (41.7)	5817 (46.8)

Abbreviation: NA, not asked (in 2015 study).

^a^ Data are from the 2015 Center for Information & Study on Clinical Research Participation Perceptions & Insights Study (all respondents).

^b^ Data are from the 2017 Center for Information & Study on Clinical Research Participation Perceptions & Insights Study (all respondents).

^c^ Question grouped with other attribute in 2015 study.

Among younger respondents, greater importance was placed on taking time off from work and having study visits conducted at their home or office when deciding whether to participate. For older respondents, the greater concern was having access to a study drug after their participation ended.

The 2017 study also found that of those considering participation in a clinical study, 8451 (68.0%) would consult their physician and 4459 (35.9%) would prefer to initiate their search for a clinical trial this way. Consulting with peers in an online patient community was identified as another key source of information, with 9192 (74.0%) indicating that they would be somewhat or very interested. The highest interest in using this information channel was observed among younger respondents (aged 18-34 years).

### Participation Experiences

The 2017 survey results included 2 indicators that clinical study participation experiences remained positive. A total of 1154 of the 2194 respondents (52.6%) who had participated in a clinical trial rated the medical care that they received during their participation as better than what they would have otherwise received, as did 1873 (85.4%) in the 2015 survey. A total of 2049 (93.4%) reported that they would be willing to participate in another study in the future, and 2010 (91.6%) reported that they would be likely to recommend participation to family and friends if it was appropriate for them. In the 2015 survey, the figures were similarly high (3085 [97.9%] were willing to participate in future trials and 2989 [94.8%] would recommend participation to family and friends).

However, in the 2017 survey, 1075 respondents (49.0%) expressed that their clinical trial participation disrupted their daily routine; this belief was particularly pronounced among younger respondents and, to a lesser degree, among minority populations (Table 5). For the 678 respondents who once qualified for a study, 94 (13.9%) decided not to move forward after reviewing the informed consent form mostly because of fear of adverse effects (27 [28.7%]) and too many study visits (23 [24.5%]).

**Table 5.** Participant Experience by Age Groups (2017)^a^

Experience	No. (%) of Respondents by Age Subgroup					
	18-34 y (n = 201)	35-44 y (n = 173)	45-54 y (n = 323)	55-64 y (n = 631)	≥65 y (n = 866)	*P* Value^b^
Somewhat or very difficult to understand consent form	42 (20.9)^c^	28 (16.2)^c^	25 (7.7)	51 (8.1)	49 (5.7)	<.001
Somewhat or very disruptive on daily routine	96 (47.8)^c^	62 (35.8)^c^	64 (19.8)	99 (15.7)	102 (11.8)	<.001
Very willing to participate again	100 (49.8)	106 (61.3)	211 (65.3)^c^	439 (69.6)^c^	566 (65.4)^c^	<.001
Overall time commitment too much	32 (15.9)^c^	20 (11.6)	29 (9.0)	53 (8.4)	48 (5.5)	<.001
Missing too much work	28 (13.9)^c^	16 (9.3)^c^	19 (5.9)^c^	35 (5.5)^c^	12 (1.4)	<.005
Study procedures at home too cumbersome	22 (10.9)^c^	15 (8.7)	19 (5.9)	34 (5.4)	34 (3.9)	<.003
Childcare or other family care cost too much	20 (10.0)^c^	11 (6.4)^c^	7 (2.2)	9 (1.4)	8 (0.9)	<.001
Slow reimbursement for out-of-pocket expenses	19 (9.5)^c^	13 (7.5)	18 (5.6)	19 (3.0)	25 (2.9)	<.001

^a^ Data are from the 2017 Center for Information & Study on Clinical Research Participation Perceptions & Insights Study (those who participated in a clinical trial).

^b^ The *z* test *P* value for age variable was corrected for type I error, and the test results were adjusted by multiplying the *P* value for each test by the *df*.

^c^ Indicates statistical significance at 95% CI compared with the other age group(s).

Overall, adoption of new convenience-enhancing technologies and services appear to be in early stages. Text messaging for visit reminders or instructions (402 [18.3%]) and electronic informed consent using a tablet (369 [16.8%]) were most commonly cited compared with smartphone apps (209 [9.5%]), wearable devices (173 [7.9%]), some or all visits at the respondent’s home or office (152 [6.9%]), and concierge services (147 [6.7%]) (eFigure 2 in the Supplement).

A total of 11 298 respondents (90.9%) to the 2017 survey agreed that it was important to receive a summary of their clinical research study results. However, only 1164 of the 2194 (53.1%) who participated in clinical trials reported ever having received them—a figure consistent with that observed in the 2013 and 2015 surveys. A total of 9020 respondents (72.6%) voiced strong interest in also seeing their individual study results.

## Discussion

The results of the 2017 CISCRP study may provide a foundation from which to build meaningful and effective patient engagement. The results also revealed several significant roadblocks: knowledge gaps among the lay public about where and how research takes place, limited physician involvement in discussing clinical trials as treatment options, and the inconveniences that patients encounter once they volunteer to participate. Public sentiments about the value and general safety of clinical research have become increasingly favorable, even in the midst of clinical trial–related tragedies periodically featured in the media.^22,23^

Many of the patient engagement initiatives noted at the beginning of this article could help ease these roadblocks, including reducing and eliminating unnecessary clinical trial procedures to simplify the protocol design; soliciting patient input into protocol design to improve protocol relevance and convenience; sending electronic alerts to physicians and nurses about clinical trials available to specific patients; reaching out to patients via community champions and patient advocacy groups; and providing ongoing support of educational initiatives designed to raise awareness and increase deeper knowledge about the clinical research process.^24^ Informing would-be volunteers disqualified from a study about why they were ineligible and helping them find a relevant alternative clinical trial might also reverse patient propensity to give up searching entirely.^25^

Public and patient education that focuses on actual experiences and actionable recommendations, particularly by individuals who have had direct experience as clinical trial participants, may be a valuable approach.^26^ Depicting clinical trial volunteers as medical heroes reinforces the altruistic gift of participation.^27^ Sharing a plain-language summary of study results after participation would maintain the engagement of study volunteers and help them better understand the value of their contribution, both of which may improve their odds of volunteering again. There have been many recent developments along these lines: US regulators have indicated that they favor the return of plain-language clinical trial results summaries,^28^ the European Medicines Agency will soon be requiring lay person summaries,^29^ and a large and increasing number of major biopharmaceutical companies have proactively committed to sharing data and disseminating plain-language results summaries to their study volunteers, in some cases with individual information, such as whether a participant received an active drug or a placebo.^30,31,32,33,34^

As has been reported,^8^ physicians are the ultimate partners with their patients: physicians are patients’ preferred source for clinical research information, and physicians are patients’ most trusted advisers when considering clinical trial participation. However, physicians responding to a recent global study^8^ reported referring only 7% of their patients to clinical trials; thus, patients interested in pursuing the option may have to do so alone if not actively discouraged from doing so. The primary reasons that physicians did not refer patients were a lack of access to information and a general lack of time, not uneasiness in starting the conversation. Most physicians (91%) were somewhat comfortable if not very comfortable discussing the opportunities. An additional benefit of better integrating clinical research and clinical care would be more effective translation of study results into clinical practice.^35^

Participating in a clinical trial is burdensome for patients, whose daily routines might be disrupted by the need to return to an investigative site multiple times for study-related procedures and on- or off-site laboratory work, particularly because protocols have become increasingly complex. Soliciting feedback from patients and investigative site staff to optimize protocol designs should help reduce participation burden.^3^ Greater adoption of convenience-enhancing initiatives and technologies may also be important in retaining clinical trial enrollees. The results of the 2017 survey indicate that smart phone apps, concierge services, and home study visits are the most desired services, none of which top the list of accommodations actually offered to participants at this time.

In developing their recruitment and retention strategies, study sponsors might pay particular attention to any negative impressions of research held by women because they are known influencers of health care decisions in the household.^36^ They also need to consider differences between patients, as a function of their age, background, health status, geographic location, and familiarity with research, so that education and engagement efforts can be customized accordingly. Younger and older individuals are differently affected by the time commitment of trial participation, for example, and their preferred way to learn about clinical research, and their views on the care that they receive in a study also vary significantly.^13,14^ People in certain regions of the world also tend to be more trusting of pharmaceutical companies but less likely to view clinical studies as safe compared with their counterparts in North America.^15^ Subsequent studies presenting the results of subgroup analysis will endeavor to uncover key trends and opportunities.

### Limitations

The results of the 2017 CISCRP survey and those of past surveys are based on responses from convenience samples. Although the number of international responses, particularly in the 2017 study, was large, the surveys were conducted online among adults who self-identify as people seeking health-related information and who have opted to receive email communications and invitations. As such, the results of these surveys should be viewed with some caution because they reflect sampling bias and may not be representative of the views of the entire global population, particularly those who cannot access, receive, and read online solicitations and communications.

## Conclusions

The results of this study may provide a foundation from which to build meaningful and effective engagement with the public and patients and reveal roadblocks, including knowledge gaps among the public, limited physician involvement in discussing clinical trials as treatment options, and the inconveniences that patients encounter once they volunteer to participate. These findings may inform patient engagement strategies and tactics and ultimately help accelerate the drug-development process.

## Notes

Supplement. **eFigure 1**. Where Trial Participants First Learned About Study **eFigure 2**. Services and Technologies Used in Clinical Trial
