# Supplementary material for: Global Public Attitudes About Clinical Research and Patient Experiences With Clinical Trials
Source: JAMA Netw Open. 2018 Oct 5;1(6):e182969. doi: 10.1001/jamanetworkopen.2018.2969 (PMC6324429; doi:10.1001/jamanetworkopen.2018.2969)
Supplement: Supplement. — eFigure 1. Where Trial Participants First Learned About Study eFigure 2. Services and Technologies Used in Clinical Trial [file jamanetwopen-1-e182969-s001.pdf]

Supplementary Online Content

Anderson A, Borfitz D, Getz K. Global Public Attitudes About Clinical Research and Patient Experiences With Clinical Trials. *JAMA Netw Open*. 2018;1(6):e182969.

eFigure 1. Where Trial Participants First Learned About Study

eFigure 2. Services and Technologies Used in Clinical Trial

This supplementary material has been provided by the authors to give readers additional information about their work.

© 2018 Anderson A et al. *JAMA Network Open.*

eFigure 1

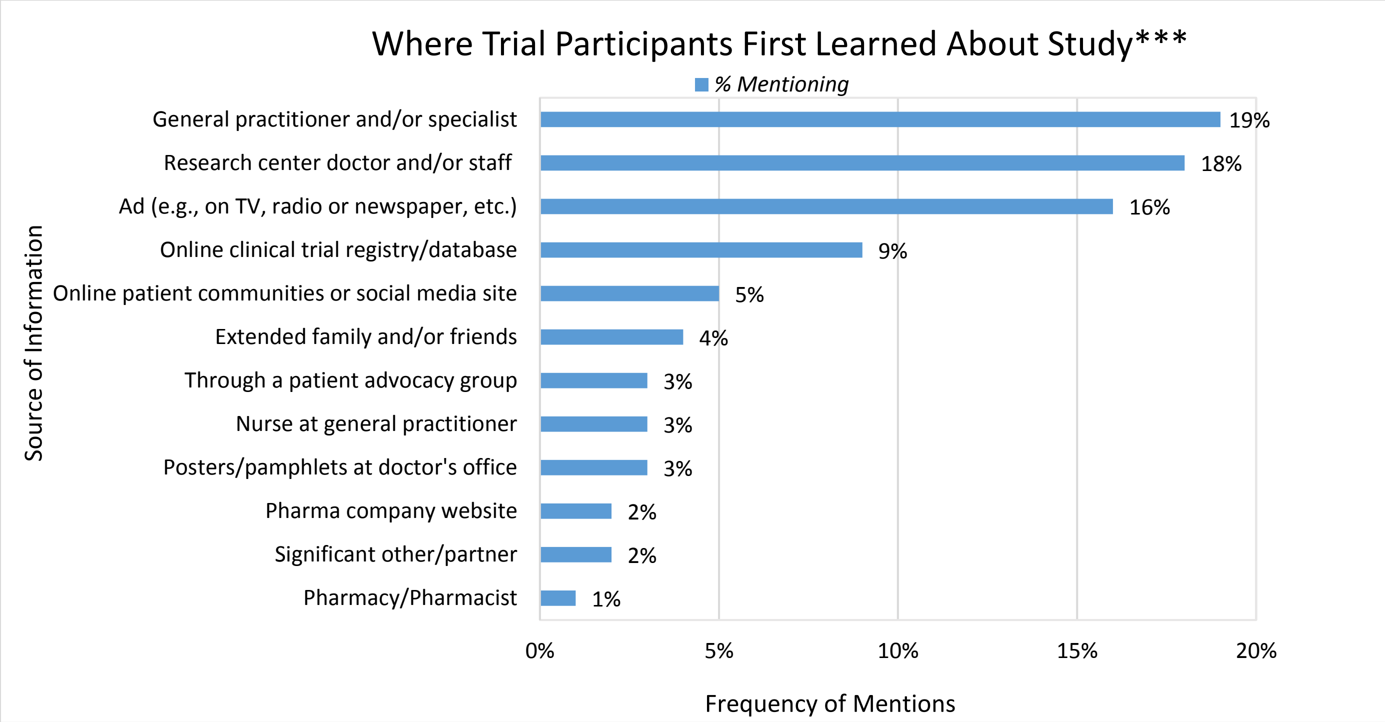

****2017 Center for Information & Study on Clinical Research Participation Perceptions & Insights Study, Those who participated in a clinical trial (n=2,194)*

© 2018 Anderson A et al. *JAMA Network Open.*

eFigure 2

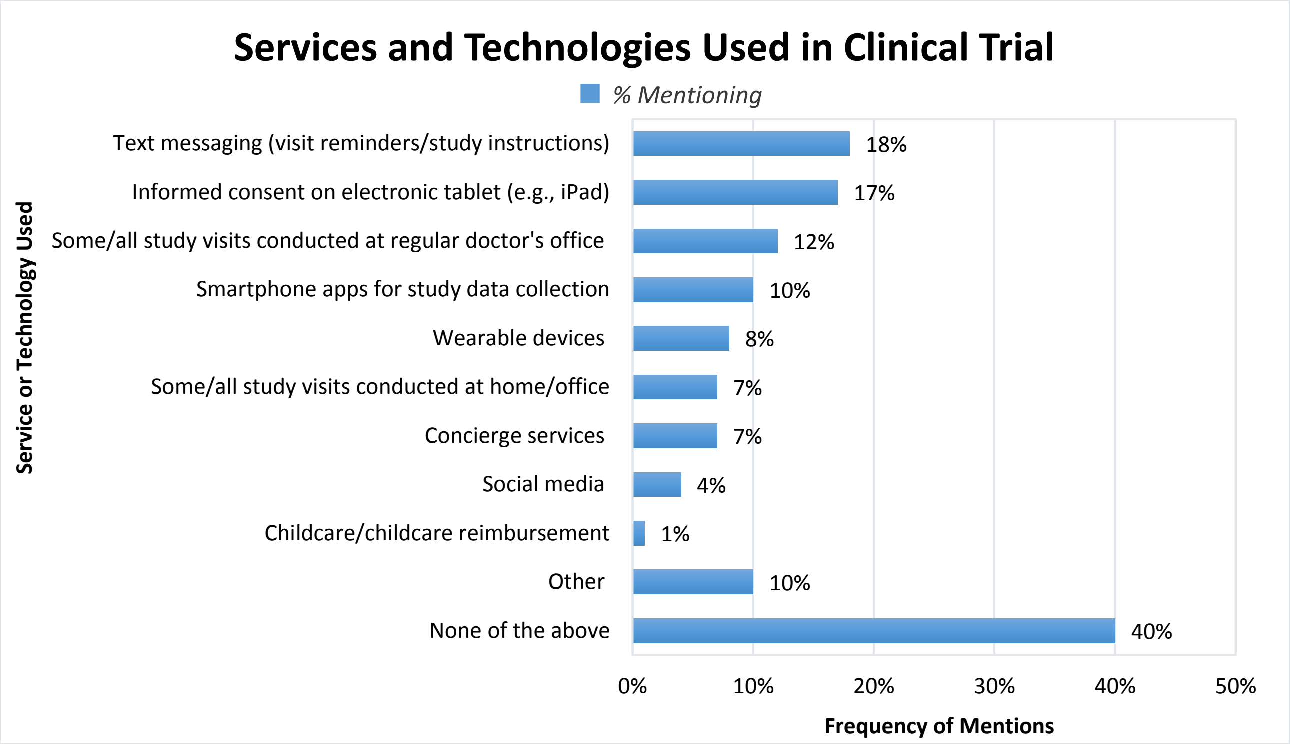

****2017 Center for Information & Study on Clinical Research Participation Perceptions & Insights Study, Those who participated in a clinical trial (n=2,194)*

© 2018 Anderson A et al. *JAMA Network Open.*
